# Neural and Self-Report Markers of Reassurance: A Generalized Additive Modelling Approach

**DOI:** 10.3389/fpsyt.2020.566141

**Published:** 2020-09-23

**Authors:** Jeffrey J. Kim, Trent Henderson, Talitha Best, Ross Cunnington, James N. Kirby

**Affiliations:** ^1^ Compassionate Mind Research Group, School of Psychology, The University of Queensland, Brisbane, QLD, Australia; ^2^ The Centre for Advanced Imaging, The University of Queensland, Brisbane, QLD, Australia; ^3^ Orbisant Analytics, Brisbane, QLD, Australia; ^4^ School of Health, Medical and Applied Sciences, Central Queensland University, Brisbane, QLD, Australia

**Keywords:** compassion, fMRI, self-report, modelling, reassurance, criticism

## Abstract

Research has shown that engaging in self-reassurance, a compassionately motivated cognitive relating style, can down-regulate neural markers of threat and pain. Whilst important, the relationship between neural and self-report markers of reassurance are largely unknown. Here we analyzed previously published fMRI data which measured neural responses when participants engaged in self-reassurance toward a mistake, setback, or failure. Within the present paper, we identified correlations between regions of interest extracted during self-reassurance with fMRI and self-report data. Using generalized additive modelling, we show that participants with greater inadequate forms of self-criticism exhibited greater neural activation within the medial prefrontal cortex (MPFC) and anterior insula (AI). Furthermore, a relationship between greater fears of expressing compassion to the self and neural activation within the MPFC returned non-significant after correction for multiple comparisons. No significant relationships were observed between brain activation and hated and reassuring forms of self-criticism. Our results identify preliminary evidence for neural activity during self-reassurance as correlated with self-report markers, and we outline a method for modelling neural and self-report data which can be applied to future studies in compassion science, particularly with a clinical sample.

## Introduction

Self-reassurance is a way of being compassionate to one’s own suffering, and has been operationalized as a way to be soothing, encouraging, and supportive to oneself in the face of setbacks (1–3). In contrast, self-criticism is considered to be a self-relating style embedded in a competitive or “rank-based” motivational system. Studies have consistently found that self-criticism, insecure striving, and fears of receiving compassion from others have all been linked to increased depressive symptoms (4–6). Importantly, this association no longer exists for individuals with higher levels of self-reassurance, suggesting a buffering effect on mental-health *via* self-reassurance (1). According to social mentality theory (7), self-reassurance is a cognitive relating style that stems from the compassionate motivational system. The function of self-reassurance is to soothe and calm the individual by activating the person’s caring/affiliative affect regulation system, also known as the “tend and befriend” or “rest and digest” system (8, 9). Accordingly, the physiological function of self-reassurance is to activate the parasympathetic system in times of perceived threat (2). When locked in a competitive or “rank-based” motivational system, as opposed to a compassionate one, individuals find self-criticism easy and self-reassurance difficult (10, 11).Typically, the function of self-criticism is to correct errors, but it can also serve a function to punish mistakes, and although both significantly predict depressive symptoms, it is the latter which is particularly powerful (3, 12).

Importantly, compassion is a motivation, which involves having a sensitivity to suffering in self and others, with a commitment to try and alleviate and prevent it (13). Cultivating a compassionate motivational system, can enable an individual to cultivate supportive and encouraging abilities and their internal supportive “physiological infrastructure” (e.g., parasympathetic system and the vagus) (14–16) in which the buffering impact of self-reassurance can work, opening the opportunity for the individual in times of distress to be helpful rather than hurtful or harmful (17). Indeed, there are now therapies developed, such as Compassion Focused Therapy (8) aimed at motivational switching to help individuals who struggle with high levels of self-criticism and shame. But how self-reassurance may work on a neural level, and how this may link to self-report data is less well known.

Within the current paper, we report on an fMRI paradigm which has been reported on previously (2, 18). This experiment investigated the neurophysiological correlates of compassionate mind training, and with fMRI, assessed participant’s neural responses when generating self-relating styles towards mistakes, setbacks and/or failures. Comparisons between activation patterns for self-critical versus self-reassuring relating styles have been reported elsewhere (2, 19, 20), The aim of the present paper is to explore how regions of the brain activated during self-reassurance may be attenuated by fears of compassion and forms of self-criticism.

## Method

### Experimental Procedure and Participants

As reported previously and reproduced in part due to a CC BY license (2), 40 participants (Mean age = 22 years, SD = .49, 27 female) were invited to engage in either self-reassuring or self-critical thoughts to stimuli which describe a mistake, setback, or failure (stimuli were counterbalanced for an emotional and neutral condition). Pre-testing on 200 participants revealed emotional statements were of sufficient intensity and negative valence as compared with neutral statements. Participants underwent 20 practice trials to test the task before the scanner, and rated the intensity of self-reassurance and self-criticism they felt for each trial *via* button-press on an MRI-compatible fibre-optic button box within the experiment. A relevant University Human Research Ethics committee approved the experimental protocol, and all participants provided written and/or digital informed, voluntary consent, and received remuneration at the value of $60 Australian dollars for participation. Whilst we observed no participant attrition across the combined fMRI experiment and HRV training as reported previously, one participant failed to complete an fMRI session due to feeling anxious during the scan, leaving us with a total number of 40 participants who completed both fMRI and HRV components. fMRI methods, pre-processing, and analysis are reported previously (2), yet regions-of-interest (ROI) extraction is summarized below for clarity:

Group-level analyses were conducted for emotional – neutral stimuli overall. As reported previously (2), brain regions shown to be significant had their anatomical labels identified with the Automated Anatomical Labelling (AAL) toolbox implemented in SPM12. Next, in order to examine correlations between the level of neural activation (i.e. difference in response between emotion verses neutral) and the mindset participants engaged in (i.e. self-criticism versus self-reassurance), we performed additional ROI analyses. For each ROI, we identified peak clusters which showed significantly greater activation overall for emotion vs neutral stimuli, and used these coordinates to extract the average contrast parameter estimates (i.e. levels of activation, Beta weights) with 5 mm radius spheres centered on those peaks for each mindset (i.e., self-criticism and self-reassurance).

#### Fears of Compassion Scale

We utilized the fears of compassion scale within the present research (5), which has three subscales; measuring fear of compassion for the self (example item, “I fear that if I become more self-compassionate I will become a weak person”), fear of receiving compassion from others (example item, “When people are kind and compassionate towards me I feel anxious or embarrassed”), and fear of compassion for others (example item, “Being compassionate towards people who have done bad things is letting them off the hook.”). Our sample comprised an internal consistency of 0.85 for compassion toward the self, 0.90 for compassion to other, and 0.85 for receiving compassion from others.

#### Forms of Self-Criticism/Self-Reassuring Scale

This 22-item scale assesses participants’ thoughts and feelings about themselves during a perceived failure. Two subscales measure forms of self-criticising (inadequate self (e.g., “I think I deserve my self-criticism”) and hated self (e.g., “I have become so angry with myself that I want to hurt or injure myself”), and one of reassurance (reassured self, e.g., “I am gentle and supportive with myself.”) (21). Our sample comprised an internal consistency of 0.89 for inadequate, 0.70 for hated, and 0.86 for reassuring forms of self-criticism, respectively.

## Results

### Correlations and GAMs

Previously we reported how activation in a few key regions of the brain (Anterior Cingulate, Amygdala, and Anterior Insula) were down-regulated when participants engaged in self-reassurance, versus self-criticism (2). Interestingly, activation across numerous regions were shown to not change their activation based on the mindset participants engaged in (Medial Pre-Frontal Cortex, Posterior Cingulate, and Lingual Gyrus). We will continue to use these same ROIs in the present paper for consistency to correlate with self-report measures (fears of compassion to self, forms of self-criticism). All combinations of brain regions and self-report variables have been reported in a table for clarity of measures (**Table 1**).

**Table 1:** Correlations between brain ROIs during self-reassurance and self-report markers.

Variable		Forms: Inadequate	Forms: Hated	Forms: Reassuring	Fears: Respond	Fears: Express to Self	Fears: Express to Other	ACC Response	AI Response	Amygdala Response	Lingual Gyrus (Visual Cortex) Response	MPFC Response	PCC Response
1. Forms: Inadequate	Pearson’s r	—											
	p-value	—											
2. Forms: Hated	Pearson’s r	0.679***	—										
	p-value	**<.001**	—										
3. Forms: Reassuring	Pearson’s r	-0.486**	-0.632***	—									
	p-value	**0.001**	**<.001**	—									
4. Fears: Respond	Pearson’s r	0.137	0.274	-0.298	—								
	p-value	0.412	0.097	0.069	—								
5. Fears: Express to Self	Pearson’s r	0.258	0.205	-0.262	0.674***	—							
	p-value	0.118	0.217	0.112	**<.001**	—							
6. Fears: Express to Other	Pearson’s r	-0.006	0.15	0.04	0.197	0.005	—						
	p-value	0.97	0.368	0.813	0.236	0.976	—						
7. ACC Response	Pearson’s r	0.208	0.206	-0.184	0.151	0.096	0.236	—					
	p-value	0.198	0.201	0.257	0.367	0.566	0.154	—					
8. AI Response	Pearson’s r	0.362*	0.267	-0.025	0.092	0.313	0.057	0.465**	—				
	p-value	**0.022**	0.095	0.878	0.581	0.056	0.734	**0.003**	—				
9. Amygdala Response	Pearson’s r	0.179	0.092	-0.157	-0.013	0.095	-0.037	0.592***	0.379*	—			
	p-value	0.27	0.572	0.335	0.936	0.569	0.827	**<.001**	**0.016**	—			
10. Lingual Gyrus (Visual Cortex) Response	Pearson’s r	0.11	0.234	0.073	0.165	0.182	0.117	0.454**	0.475**	0.426**	—		
	p-value	0.498	0.146	0.657	0.324	0.273	0.483	**0.003**	**0.002**	**0.006**	—		
11. MPFC Response	Pearson’s r	0.379*	0.15	-0.278	0.155	0.363*	-0.035	0.218	0.433**	0.189	0.109	—	
	p-value	**0.016**	0.356	0.083	0.353	**0.025**	0.834	0.177	**0.005**	0.242	0.503	—	
12. PCC Response	Pearson’s r	0.329*	0.108	-0.201	0.101	0.212	0.079	0.29	0.581***	0.308	0.011	0.649***	—
	p-value	**0.038**	0.509	0.214	0.545	0.202	0.639	0.07	**<.001**	0.053	0.945	**<.001**	—

Bold text indicates a significant relationship. *p < .05, **p < .01, ***p < .001.

**Figure 1:**
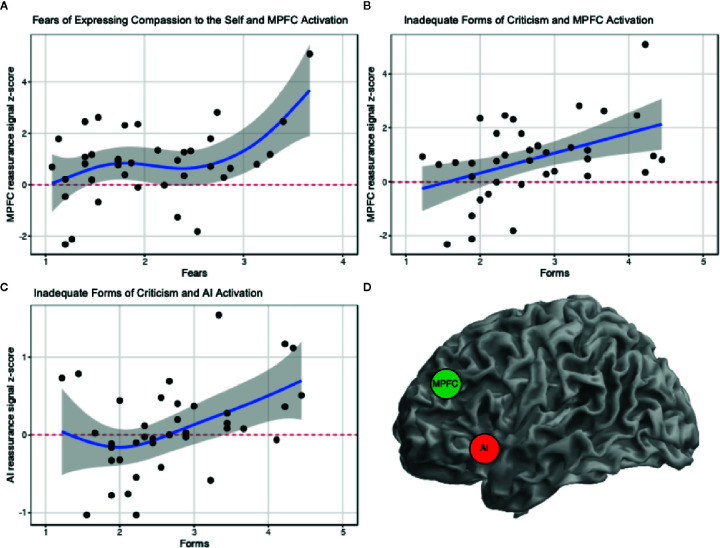
GAM results of significant correlations. **(A)** MPFC ~ Fears: Smooth function for fears of expressing compassion to self significantly predicts MPFC reassurance signal. **(B)** MPFC ~ Forms: Smooth function for inadequate forms of criticism significantly predicts MPFC reassurance signal. **(C)** AI ~ Forms: Smooth function for inadequate forms of criticism significantly predicts AI reassurance signal. **(D)** Rough spatial location of MPFC (2 46 36) and Left AI (−26 10 −14) ROIs. Coordinates reported in MNI-space.

Our analysis plan proceeds as follows; first, we will explore the relationships (correlations) between all ROIs and self-report measures. Second, we will use GAMs to model the significant relationships. As can be seen in **Table 1** and **Supplementary Figure 1**, an initial correlation analysis revealed relationships with fears of self-compassion and inadequate forms of self-criticism in the MPFC and AI. Furthermore, also shown in **Table 1** and depicted in **Supplementary Figures 2, 3**, non-significant relationships of reassuring and hated forms of criticism with MPFC and AI are reported. To follow-up the significant relationships observed for inadequate forms of criticism and brain responses, three generalized additive models (GAM) were used to test statistically the relationships between explanatory (i.e., self-report) and response variables (brain activation: ROIs). Candidate variables for modelling with GAMs were selected after inspection of correlations **(** **Table 1** **)** and scatterplots (**Supplementary Figures 1–3**) revealed relationships which would benefit from the smoothing functions implemented under GAMS. Indeed, GAMS are similar to General Linear Model (GLMs), however in GAMs, smooth functions for each covariate are added (22, 23) in circumstances where traditional linear models are insufficient. Basis functions in GAMs are summed (connected) using spline interpolation, typically with a polynomial (22, 23). A benefit of GAMS is the provision of model evidence: here, GAMS can go beyond correlations to examine how well a model may fit data which is not strictly linear, and can contribute p-values, as well as R2 and deviance explained (similar to unadjusted R2, when the link function is Gaussian). As can be seen in **Figure 1** and **Tables 2**, **3**, GAMS can appropriately model the neural and self-report data. However, note that upon multiple comparison correction (0.05/3 = 0.016), the relationship between MPFC activation and Fears of Expressing Compassion to the Self returned non-significant. We also conducted GAMs on hated and reassuring forms of criticism, however hated forms violated a degrees of freedom assumption (possible due to a floor effect in a non-clinical sample), and reassuring forms returned a non-significant GAM (**Supplementary Tables 1, 2**).

**Table 2:** Model comparison and significance of GAMS for each self-report variable and ROI.

Model	edf	ref.edf	*F*	*p*
MPFC ~ Fears	3.21	3.99	3.24	*p* = .023
MPFC ~ Forms	1.00	1.00	8.87	*p* = .005
AI ~ Forms	3.58	4.42	3.35	*p* = .018

Tilde (~) indicates the regression of Y-variable (brain variable) on X-variable (self-report score). Edf indicates estimated degrees of freedom. Ref.edf indicates reference degrees of freedom. p indicates frequentist statistical thresholded. Note that upon multiple comparison correction (0.05/3 = 0.016), the relationship between MPFC activation and Fears of Expressing Compassion to the Self returned non-significant.

**Table 3:** Deviance and variance explained of GAM models.

Model	*n*	Deviance explained	GCV	Scale estimate	Adj. *R* ^2^
MPFC ~ Fears	38	31.0%	1.73	1.54	0.24
MPFC ~ Forms	38	19.8%	1.77	1.68	0.18
AI ~ Forms	38	33.3%	0.27	0.24	0.26

Tilde (~) indicates the regression of Y-variable (brain variable) on X-variable (self-report score). Deviance explained is comparable to unadjusted R2. GCV is the minimized generalized cross-validation score of the fitted GAM—an indicator of the smoothness function. Scale estimate is the value of Φ (Phi) estimated during model fitting. Adjusted R2 is the unbiased estimate of the population R2, or variance explained by the model.

## Discussion

Within the present research we explored relationships between neural markers of self-reassurance and fears of compassion and forms of self-criticism. Using the ROIs outlined in the previous paper (2), we used GAMs to fit the extent of neural activation within each ROI from self-report markers of self-reassurance’s inhibitors (fears of compassion and forms of self-criticism). An initial correlation matrix followed-up with GAMs revealed significant relationships between neural markers of self-reassurance during fMRI and fears of compassion and forms of self-criticism (**Figure 1**). Specifically, participants with greater fears of expressing compassion toward the self, have greater neural activation within the MPFC when engaging in self-reassurance (**Figure 1A**) (however, a correction for multiple comparisons returned non-significant). In addition, participants with greater inadequate forms of self-criticism were shown to have greater neural activation within the MPFC and AI (**Figures 1B, C**).

### Implications of Results

First, the MPFC is a node in the default-mode network, which is involved with mentalizing and self-referential thoughts (24). Previous work has linked alterations in DMN function within major depression, particularly through increased functional connectivity of these regions with other neural networks such as the salience network and central executive (attention) networks (25–28). Indeed, that we observed both increased MPFC (**Figure 1B**) as well as AI response (**Figure 1C**) may imply that inadequate forms of self-criticism is an index of functional co-activation of these regions. Accordingly, from the DMN and depression literature described above, it is possible that we are identifying within a healthy control sample a subset of participants who might be tending towards depressive-like rumination, as indexed from greater self-report inadequate forms of criticism. However, future work with a larger sample, and with a between participants approach (i.e., clinically depressed versus healthy controls), would need to test this theory *a priori* with a measure of functional connectivity of these brain regions (MPFC, AI).

Second, the AI is a node within the salience network, responsible in-part for processing salient negative events within the environment. This region has been reported extensively within the empathy and compassion neuroscience literature to date (29–31). What is interesting, however, is that greater activation of this region during engagement in self-reassurance, can be indexed from greater levels of inadequate forms of self-criticism. What this self-report scale measures is the degree to which an individual feels inadequate and inferior, and therefore their self-criticism is used to correct and improve the self, in order to try harder, to achieve goals and to maintain certain standards (32, 33). Accordingly this relationship might indicate that participants with greater scores on this self-report scale might actually be “beating themselves up”, importantly, “for their perceived own good” for the imagined mistakes, setbacks or failures so that they prevent them happening again (34), as may be seen in the increase in activation of the AI. However, whilst inferring mental states from fMRI data as ‘reverse inference’ has been criticized (35), future work with an MVPA machine-learning approach might be able to decode the mind-states of participants when engaged in this paradigm (35–37).

### Limitations and Future Directions

That we did not find significant relationships between hated and reassuring forms of self-criticism and brain markers are also interesting. As can be seen in **Supplementary Figures 2, 3**, the correlation between reassuring and hated forms of criticism and brain activation was non-significant. However, inspection of the scatterplots of these scales revealed a floor effect for hated forms, and a ceiling effect for reassuring forms. That we found these results can speak to aspects of our sample, namely, within a healthy population is that our sample tended toward greater self-reassuring, and less hated forms of self-criticism, which has also been found previously (12). Indeed, non-clinical samples have been shown to rate the hated-self subscale at floor (12). Yet it is curious how inadequate forms of self-criticism was associated with neural responses when engaged in self-reassurance. Future work perhaps with a clinically depressed sample to examine self-reassurance in comparison to a healthy control sample, might reveal a relationship between hated forms of criticism and brain activity when using the GAM method.

A possible candidate for the links between self-reassurance and inadequate self-criticism might be at the core of each self-relating style is it aims to regulate in order to correct behaviour and prevent bad things happening in the future. Some have drawn parallels to an inner dominant-subordinate relationship, which is developed to ensure one stays focused and does not become arrogant and disliked by others (38). However, when it comes to self-hatred criticism, this is a relating style that aims to destroy or eliminate. Some have argued it is an internal relating style that regards oneself as an enemy or an out-group. In this instance submissiveness and appeasement (a result of inadequate self-criticism) may not work at mitigating the attack (38). Rather the self-hatred attack aims to remove the “toxin”. Indeed, self-hatred and wanting to remove aspects of the self has been found in those who self-harm (38).

An extension of our results would be to conduct an fMRI paradigm which measures neural responses to self-hatred, as well as self-reassurance, generated toward aspects of the self that are liked versus disliked. Indeed, it would be fascinating to run this experiment in populations such as those with high self-hatred to aspects of self, such as body dysmorphia, body-weight shame, or even in populations of eating disorders (39–42) and to examine if interventions such as Compassion Focused Therapy, particularly designed to work with these populations (40, 43) may help attenuate these neural responses. Specifically, we propose that the same neural mechanisms which code for “neural pain” differently for in-groups and out-groups, may likewise by stimulated when considering aspects of the self that are hated. For example, research has examined the effects of neural responses to pain, particularly within the Anterior Cingulate Cortex (ACC), when viewing videos of one’s own race versus another race in suffering (44). Whilst a typical neural bias toward processing own versus other race was evident, activation to other race’s pain within the ACC significantly increased upon greater levels of physical contact with the outgroup, thereby tuning one’s own ACC to respond to the other group’s pain as if they were an ingroup (44). We hypothesize that a clinical sample with high self-report hatred to aspects of the self, would replicate this effect of suppressing neural empathy for pain toward outgroups, to the exact parts of the self that they hate, wish to get rid of, destroy, and expel (34). Critically, we also suggest that a Compassion Focussed Therapy intervention (45) would help to reduce this neural suppression, and allow processing of what was once hated as now something to incorporate as “ingroup”—as part of the self.

## Data Availability Statement

The raw data supporting the conclusions of this article will be made available by the authors, without undue reservation.

## Ethics Statement

The studies involving human participants were reviewed and approved by The University of Queensland Health and Behavioural Sciences, Low & Negligible Risk Ethics Sub-Committee. The patients/participants provided their written informed consent to participate in this study.

## Author Contributions

JJK, RC and JNK conceived and designed the initial experiment. JJK and TH conducted data analyses. JJK, TB, TH and JNK wrote the paper.

## Funding

JNK was supported by a University of Queensland Research and Teaching Fellowship. JJK was supported by an Australian Postgraduate Scholarship.

## Conflict of Interest

The authors declare that the research was conducted in the absence of any commercial or financial relationships that could be construed as a potential conflict of interest.
