# Supplementary material for: Neural and Self-Report Markers of Reassurance: A Generalized Additive Modelling Approach
Source: Front Psychiatry. 2020 Sep 23;11:566141. doi: 10.3389/fpsyt.2020.566141 (PMC7538506; doi:10.3389/fpsyt.2020.566141)
Supplement: Supplementary file 1 [file Table_1.docx]

Supplementary Information for

**Neural and self-report markers of reassurance: A generalized additive modelling (GAM) approach**

*Jeffrey J. Kim^1^*, Trent Henderson^2^, Talitha Best^3^, Ross Cunnington^1^ & James N. Kirby^1^*

^1^School of Psychology, The University of Queensland, Brisbane, Queensland, Australia

^2^Orbisant Analytics, Brisbane, Queensland, Australia

^3^School of Health, Medical and Applied Sciences, Central Queensland University, Brisbane, Queensland, Australia

* Correspondence:
Corresponding Author
Jeffrey.kim@uqconnect.edu.au

**This PDF file includes:**

Figure S1: Significant brain and self-report correlations.

Figure S2: Non-significant correlations: reassuring forms of criticism and brain.

Figure S3: Non-significant correlations: hated forms of criticism and brain.

Table S1: Non-significant GAM model of reassuring forms and brain activation.

Table S2. Further model output of reassuring forms and brain activation.


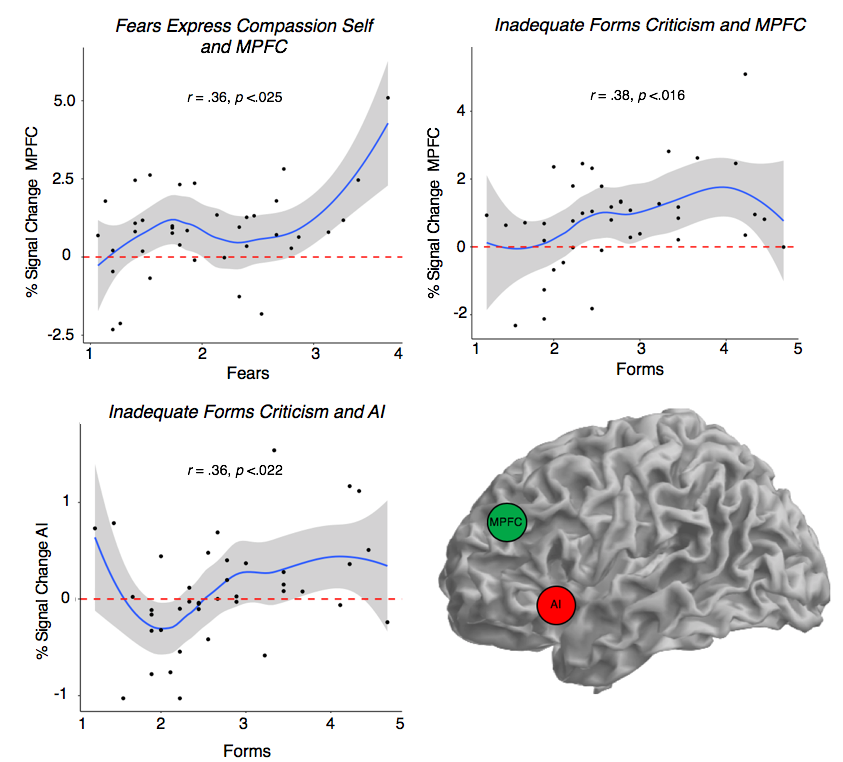


***Figure 1.*** Significant correlations between self-report scales and brain response.


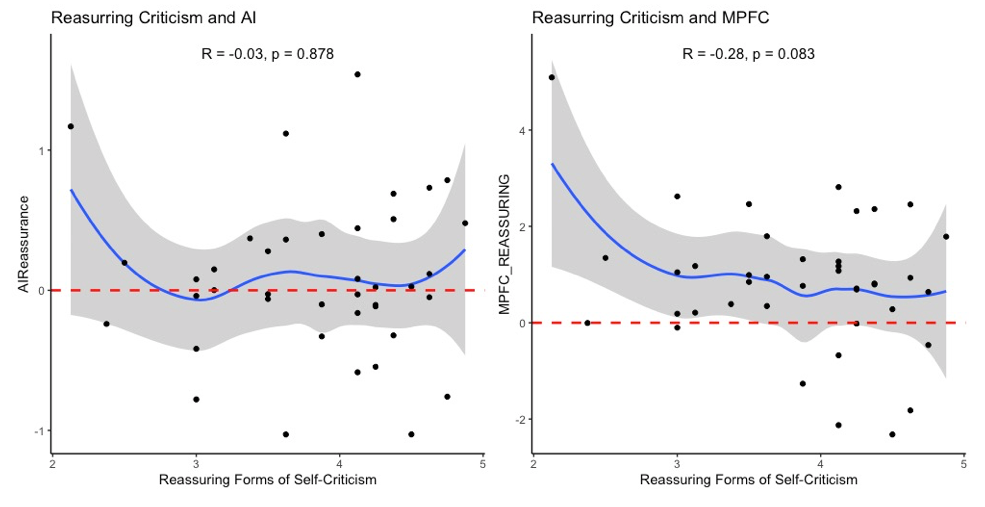


***Figure 2.*** Non-significant correlations between reassuring forms of criticism and brain response.


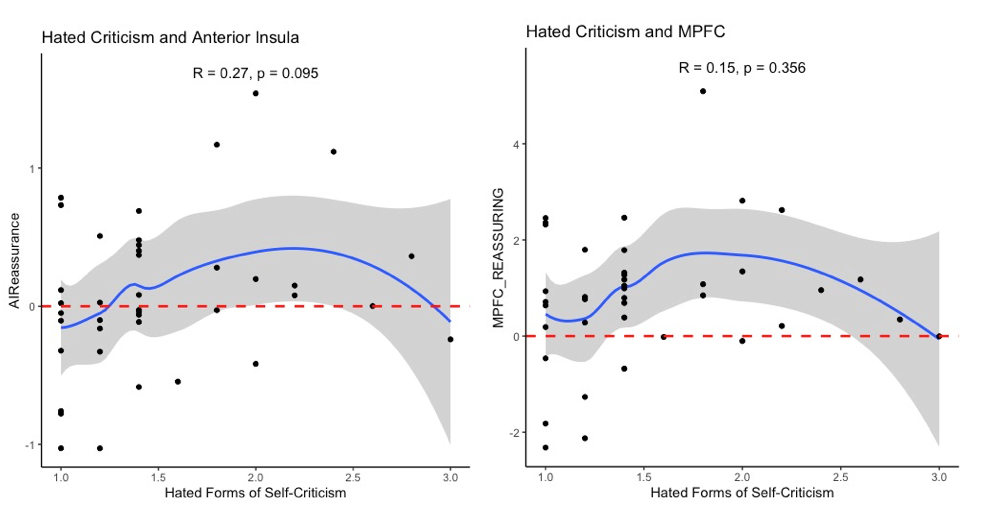


***Figure 3.*** Non-significant correlations between hated forms of criticism and brain response.

| Model | edf | ref.edf | *F* | *p* |
| --- | --- | --- | --- | --- |
| MPFC ~ Reassurance Self-Criticism | 1 | 1 | 0.30 | *p* = .592 |
| AI ~ Reassurance Self-Criticism | 1 | 1 | 0.23 | *p* = .639 |

***Table S1.*** Non-significant GAM model of reassuring forms and brain activation.

| Model | *n* | Deviance explained | GCV | Scale estimate | Adj. *R*^2^ |
| --- | --- | --- | --- | --- | --- |
| MPFC ~ Reassurance Self-Criticism | 18 | 1.4% | 2.08 | 1.85 | -0.04 |
| AI ~ Reassurance Self-Criticism | 18 | 1.4% | 0.45 | 0.40 | -0.05 |

***Table S2.*** Further model output of reassuring forms and brain activation.
